# Indigenous knowledge around the ethics of human research from the Oceania region: A scoping literature review

**DOI:** 10.1186/s13010-021-00108-8

**Published:** 2021-10-09

**Authors:** Etivina Lovo, Lynn Woodward, Sarah Larkins, Robyn Preston, Unaisi Nabobo Baba

**Affiliations:** 1 College of Medicine, Nursing and Health Sciences, Fiji National University, Suva, Fiji Islands; 2 James Cook University, Townsville, QLD 4811 Australia; 3 School of Health, Medical and Applied Sciences, CQUniversity Australia, 538 Flinders St, Townsville, QLD 4810 Australia; 4 College of Humanities and Education, Fiji National University, Nasinu Campus, Suva, Fiji Islands

**Keywords:** Ethics, Oceania, Pacific, Indigenous research ethics principles, Values, Human research ethics committees

## Abstract

**Background:**

Many indigenous people have died or been harmed because of inadequately monitored research. Strong regulations in Human Research Ethics (HRE) are required to address these injustices and to ensure that peoples’ participation in health research is safe. Indigenous peoples advocate that research that respects indigenous principles can contribute to addressing their health inequities. This scoping literature review aims to analyze existing peer reviewed and grey literature to explore how indigenous values and principles from countries of Oceania are incorporated into HRE and the governance of research involving human participants.

**Methods:**

A scoping literature review framework was used for this study. A search for peer reviewed and grey literature from Google, bibliographies, and electronic databases such as SCOPUS, SPRINGER, Medline (Ovid) and JBI Database of Systematic Reviews was conducted, limited to the years 2002–2020. Sixty (60) documents that focused on indigenous knowledge from Oceania region and HRE were included, from which key findings and themes were synthesized.

**Results:**

Charting the data showed that more than half the eligible documents were peer-reviewed journal articles (54%). Other sources included: International Declarations on Human Research (8%); book chapters (8%); government documents (8%); HRE Guidelines or protocols (13%); news articles (7%) and PhD thesis (2%). The literature was from Australia, Cook Islands, Guam, New Zealand, Fiji, Samoa, Tonga and Vanuatu, some of which focused specifically on HREs in the Pacific Region. Issues emerging from the literature were grouped into five themes (i) indigenous and cultural principles of HRE; (ii) informed consent in indigenous settings in Oceania; (iii) vulnerability and minority status of indigenous populations exploited for research; (iv) research ethics governance for Oceania indigenous peoples; and (v) research ethics committees in Oceania. Respect, relationship building, and trust were priority indigenous HRE principles that encompass the principles of partnership, capacity building, reciprocity, and equality. Relationship building and trust imply the equal distribution of benefits for indigenous population and researchers.

**Conclusion:**

Indigenous principles of HRE identified were interconnected and interdependent. Recommendations were to incorporate indigenous principles of research in HRE regulations and processes of all countries with indigenous populations. This is especially pertinent for emerging national research committees in LMIC countries, including Fiji and Tonga. Relationship building among researchers and indigenous populations is key to successful research with indigenous populations. HRE principles important for relationship building include respect that is reciprocal among researchers and indigenous people. Elements of the principle of respect highlighted are empathy, collaboration, sharing of benefits, reciprocity, appreciation, empowerment, protection, safety and awareness of culture and languages. Indigenous ontology from the Oceania region involves spirituality, connectedness to land, religious beliefs and a participatory approach to HRE and should be respected in research. An ethical governance mechanism of HRE is one that incorporates indigenous principles and applications for the purpose of maximizing the protection of the dignity and rights of indigenous peoples of Oceania.

## Introduction

Human Research Ethics aims to ensure that research is conducted to the highest ethical standard and that human participants in research are protected [97]. Important statements of ethical principles involved in human research such as the Nuremberg Code [75], the Declaration of Helsinki [98] and the International Ethical Guidelines For Health-Related Research Involving Humans [13], have provided guidance for the governance of HRE activities. This applies in countries of Oceania [8] including Australia, New Zealand, Fiji and Tonga, as stipulated in their National Guidelines for the ethical conduct of research involving human beings, [28, 41, 65].

Thousands of people have lost their lives [67, 68] and many others have been harmed through research and medical experimentation [4, 47, 49]. These negative consequences fall disproportionately on the disadvantaged [4] and indigenous population groups [4, 59]. Two significant events have highlighted the deficiencies in human research governance mechanisms in research institutions [4, 67]. One of these was the Nuremberg Trials in 1945–1946 when Nazi doctors were brought to trial for conducting experiments on thousands of prisoners resulting in their deaths during World War II. The second was the 40-year-long study of black American men with untreated syphilis in Tuskegee Alabama, 1932–1972 [4]. The Tuskegee study sparked outrage in 1972 after it became widely known that despite the discovery of penicillin as a cure for syphilis, the participants in the Tuskegee study were not treated. Many were unaware they were in a study and many were harmed and died as a result [14, 79].

These deficiencies prompted calls for strong regulations in health research ethics to ensure that people participating in health research are not harmed [13, 21, 67, 83, 98]. HRE regulations were formed in countries like Australia [65], Canada [10], New Zealand [62], Fiji [28, 80], as they responded to the need to strengthen governance mechanisms for the protection of human participants in research. HRE systems exist in a few Pacific Island Countries (PICs) in the Oceania region [28, 80] but are non-existent in others like Tuvalu and Kiribati. Indigenous populations are frequently researched [24] but their own values and practices that should guide research involving themselves are not always made known to researchers. Therefore national HRE must consider the unique cultural values and practices and include these in national guidelines for HRE [83] in order to guide researchers to conduct research appropriately in indigenous populations.

Table 1 presents the chronology of these advances in HRE in Oceania and internationally.

**Table 1:** Chronology of International Organizations and Oceania Region HRE Advances for the Protection of Human Participants in Research

INTERNATIONAL		OCEANIA REGION	
Year	Report Titles	Year	Report Titles
1947	The Nuremberg Code: International [82]	2003	Values and Ethics: Guidelines for Ethical conduct in Aboriginal and Torres Strait Islander Health Research [66]
1948	The International Declaration of Human Rights by the United Nations General Assembly [87]	2010	Te Ara Tika: Guidelines for Māori research ethics: [38]
1964	The Declaration of Helsinki by the World Medical Association [97]	2011	The University of Otago, Pacific Research Protocols (2011) [5]
1966	International Covenant on Civil and Political Rights [69]	2014	Health Research Council’s Pacific Health Research Guidelines (2014) [39]
1978	The Belmont Report: Ethical principles and guidelines for the protection of human subjects of research [89]	2014	Tonga Ministry of Health. Operational Guidelines for the National Health Ethics and Research Committee [81]
2000	WHO Operational Guidelines for Ethics Committees that Review Biomedical Research [95]	2015	National Health Research Guide [30]
2005	Council of Europe. Additional Protocol to the Convention on Human Rights and Biomedicine, concerning Biomedical Research [14]	2015	Centre for Samoan Studies, University Research Ethics Committee, National University of Samoa [11]
2006	UNESCO Universal Declaration on Bioethics and Human Rights [84]	2017	Pacific Research Guidelines and Protocols, Pacific Research & Policy Centre and the Pasifika@Massey Directorate, Massey University [55]
2007	United Nations declaration on the rights of indigenous people		
2008	UNESCO Bioethics Core Curriculum [85], and the United Nations Declaration on the Rights of Indigenous Peoples [86]	2018	Ethical conduct in research with Aboriginal and Torres Strait Islander Peoples and communities: Guidelines for researchers and stakeholders 2018 [65].
2011	WHO Western Pacific Region: The Ethics Review Committee Standard Operating Procedures for Ethics Review Committee of the WHO Regional Office for the Western Pacific [96]	2020	Fiji Human Health Research Policy [29].

### Indigenous HRE

Indigenous is defined as “people who are descendants from populations that inhabited the region before the time of conquest and colonization, and who, independently of their legal status, have preserved all or part of their social, economic, cultural and political institutions, and that, at the same time, self-recognize themselves as such” ([58], p.201).

The Oceania Region is divided into four major sub-regions; (i) Australia and New Zealand sub-region include Australia, Christmas Island, Cocos Islands, Heard Island, New Zealand and Norfolk Island, (ii) Melanesia sub-region include Fiji, New Caledonia, Papua New Guinea, Solomon Islands, and Vanuatu, (iii) Micronesia include Guam, Kiribati, Marshall Islands, Nauru, Northern Mariana Islands and Palau, (iv) Polynesia sub-region include Pitcairn Islands, Samoa, Tokelau, Tonga, Tuvalu and Wallis and Futuna [56, 88].

Indigenous people of the Pacific Islands are divided into three main groups, Melanesia, Micronesia and Polynesia [43]. Melanesian indigenous populations are the iTaukei people of the Republic of Fiji [60], the Kanak of New Caledonia, the people of Papua New Guinea, the NiVanuatu of the Republic of Vanuatu [89] and the Solomon Islanders [52]. Some Polynesian indigenous people include the Cook Islanders, Maori of New Zealand, Samoan and Tahitian people [43]. Some Micronesian indigenous populations include IKiribati, Palauans and Tuvaluans [31]. For the purpose of this review, the term indigenous includes cultural knowledge from island countries of Oceania who do and do not consider themselves indigenous like Tonga.^1^ Whilst HRE systems exist in a few Pacific Island Countries (PICs) in Oceania region they are non-existent in others. The significance of focusing this review on indigenous peoples’ values and principles in HRE in the Oceania region is to highlight the need to incorporate indigenous values and principles in the governance mechanisms of research that involve them [83]. Human Research Ethics in indigenous populations are to be informed by their indigenous principles and values for relevance and applicability [83]. The involvement of indigenous paradigms and ontology in the governance of HRE has been overlooked. The international guidelines from the World Medical Association have been applied in HRE with indigenous populations without consideration of indigenous contextual relevance. Whilst HRE systems exist in a few Pacific Island Countries (PICs) in Oceania they are nonexistent in others. The aim of this review is to explore how indigenous values and principles from countries of Oceania are incorporated into HRE and the governance of research involving human participants. This study is focusing on HRE in Oceania region, due to the need to advance HRE in countries of Oceania.

## Methods

### Review methodology

A scoping literature review framework defined by Arksey and O’Malley [2] was used for this study; a common method for reviewing a broad range of literature and synthesizing research evidence particularly when few rigorous research studies address the question [14, 17, 63]. This framework involves five stages presented in the next section: (1) identifying the research question; 2) identifying relevant studies; 3) study selection; 4) charting the data; and 5) collating, summarizing and reporting the results. The scoping study process is non-linear and the researcher can repeat certain steps or check searches or tasks required to conduct a thorough search [2]. The Arksey and O’Malley framework enables the researcher to utilize a methodical system to find and collect relevant documents then summarize, consolidate and map out concepts and themes, then linked back to the research topic of international indigenous knowledge and the ethics of human research. Conclusions are drawn by synthesizing how activities of indigenous HRE are reported in the literature, including the status of activities of Indigenous HRE in the literature. The implementation of the scoping literature review is summarized below.

### Implementing the scoping literature review

**Fig. 1:**
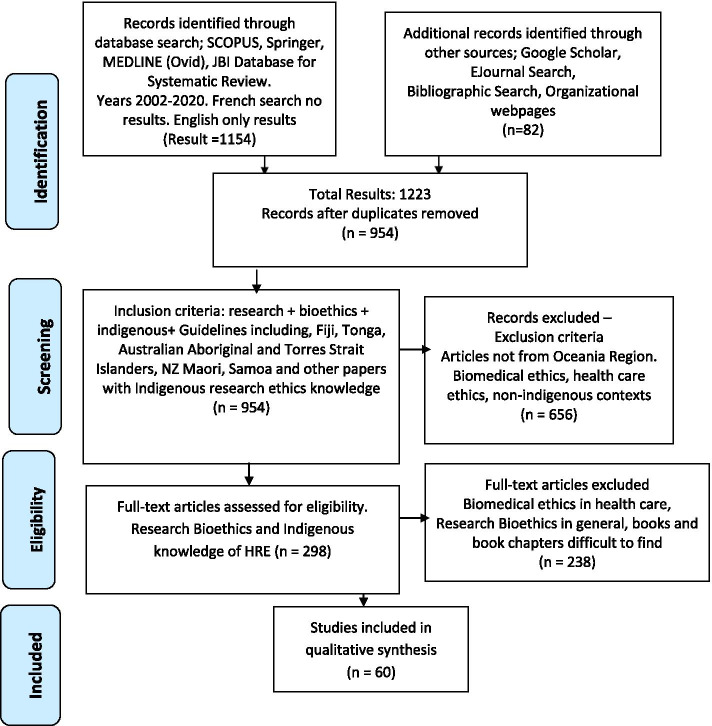
Prisma flow chart of literature search

The research questions for this scoping review were: (i) what are the existing underlying theoretical principles that inform indigenous ethical approaches of HRE? and (ii) how are indigenous principles applied in the governance of human research? The literature search process is shown in the ‘Preferred Reporting Items for Systematic Reviews and Meta-Analysis’ (PRISMA) diagram (Fig. 1) [72] and was limited to the years 2002–2020. 23rd November, 2020 was the last day of the literature search. English word search and French translation of the same words plus the names of the three French Territories in Oceania, Tahiti, New Caledonia and Wallis and Futuna were used for the search in the databases. Table 2 presents a summary of the search words.

**Table 2:** Key words in three categories

Categories	Key Words
**Ethical or Moral Principles**	“Principles Research? Ethic* Theor* OR Moral* OR “Virtue” OR “Research” AND “Bioethic*” OR “Research” AND “Morality” OR “Moral*” OR “Values” OR “beneficence” OR “Justice “OR “Non-maleficence” OR “Respect for autonomy” Or “Philosophy” AND “Research Ethics” OR “Privacy” OR “Confidentiality” OR “Informed Consent” OR “Vulnerable”
**Research Bioethics and Governance**	“Research?Ethic* Committee” AND Pacific” OR “Research? Ethic* Committee” AND “Indigenous “OR “Pacific” OR “Research? Ethics Codes” OR “Institutional Review Board” OR “Pacific Research Ethics Codes” OR “Research Ethics Guidelines”
**Indigenous HRE**	“De-colonizing research methods” OR “Pacific research? ethic*” OR “Fiji Research? ethic*” OR “Tonga Research? ethic*OR “Pacific indigenous research” OR “indigenous research? ethic*” OR “Pacific indigenous research”
**French Language Search (targeting any publication from French Territories in Oceania Region)**	*“Ethique de la recherche”* OR *“ethics committee”* OR *“comite de deontologie”* OR *“commission ethique”* AND *“indigenous”* OR *“indigène”* AND *“Tahiti”* OR *“New Caledonia” or “Wallis and Futuna”*

The first author (EL) collated the data, read and summarized all documents according to the following characteristics and recorded the following in a table; profile of document, study location, study population, aim, research ethics/bioethics principles or theories, methodology (only research articles), important results and outcomes. A framework was developed for a consistent approach to the analysis of the findings from the gathered literature. Based on the aim, the framework consisted of two categories: (i) the indigenous knowledge incorporated into HRE; and (ii) the regulations of HRE in indigenous populations.

#### Key words search

The search strategy is presented in Table 2.

The PRISMA Identification stage involved a database search in SCOPUS, Springer, MEDLINE (Ovid), JBI Database for Systematic Review and it was decided that the search will be filtered by years, 2002–2020. English search words were utilized for the search and this search yielded a total of 1154 documents. Google search was used to search for the French translation of the English search words. Then the names of the French nations in the Oceania region Tahiti, New Caledonia and Wallis and Futuna were added to the search words and a search was conducted in the databases. This search did not yield any results.

Further search for documents that may not have been found in the databases was also conducted in Google Scholar, EJournals, Bibliographic Search and in relevant Organizational webpages and 82 documents were found and the total was 1223 documents. The duplicates were removed and 954 documents remained. The next stage was the screening stage which is the strategic selection of documents to be included or excluded. Inclusion criteria strategy was to include all documents that were about research, bioethics and indigenous research ethics knowledge in countries of Oceania and exclude documents about biomedical ethics, health care ethics, about non-indigenous contexts and HRE in countries outside of Oceania region. Six hundred fifty-six documents were excluded and 298 documents remained that met the inclusion criteria. A further screening exercise was conducted where an exclusion criteria was set to exclude documents that was about Biomedical ethics in health care, Research Bioethics in general, books and book chapters difficult to find. This screening exercise resulted in the exclusion of 238 documents. The next stage required the screening of documents for eligibility. Only documents that included research bioethics and indigenous knowledge of Human research ethics were included. Documents that were excluded were about biomedical ethics in health care, research bioethics in general, books and book chapters that were difficult to find. Sixty documents remained and were included in the scoping literature review. The search process is presented as a PRISMA flow chart; Fig. 1.

## Results

The scoping literature review found that although high income countries such as Australia and New Zealand have HRE regulations that engage indigenous HRE knowledge, many low and middle income countries like Fiji and Tonga have not yet developed HRE regulations that incorporate their cultural values or indigenous values. The English search yielded results while the French search did not yield any results. Some books and book chapters could not be located or accessed, so they were not included.

Charting the data showed that more than half the eligible documents were peer-reviewed journal articles (54%). Other sources included: International Declarations on Human Research (8%); book chapters (8%); government documents (8%); HRE Guidelines or protocols (13%); news articles (7%) and PhD thesis (2%).

The geographical distribution of the literature from Oceania region were as follows; Australia 12%, Cook Islands 2%, Fiji 6%, Guam 2%, General 22%, New Zealand 18%, Pacific Islands 18%, Samoa 6% and Tonga 8%, Vanuatu 6%.

The themes that emerged were divided into five main categories: (i) indigenous and cultural principles of HRE; (ii) informed consent (IC) in indigenous settings in Oceania; (iii) vulnerability and minority status of indigenous populations exploited for research; (iv) research ethics governance for indigenous peoples; and (v) research ethics committees in Oceania. Indigenous from here onwards means indigenous knowledge from the Oceania region only. Each theme is summarized and presented in Table 3 and discussed in more detail below.

**Table 3:** Key categories and themes of Oceania indigenous knowledge reflected in the literature

(i) **Informed Consent (IC) in Indigenous Settings**	
• **Elements of IC** are respect of dignity and autonomy of persons, transparent, un-intrusive, free of coercion, free and informative, protection of human rights and bioethics, collaborative and establishing a trusting relationship [5, 32].	
• **Culturally and linguistically** appropriate [25, 40, 60].	
• **Communitarian approach to IC** [9, 32, 48, 54]	
• **Acceptable processes of IC for increased understanding;** audiovisuals and graphics, provide options of oral or written forms. Read out loud then consent can be audio recorded. Conduct IC in the local language. IC may include family members or community [32, 48, 50]	
(ii) **Indigenous Principles of HRE**	
• **Relationship building** between researchers and indigenous participants should employ the values of respect and empathy, [26, 40], collaboration, sharing of resources, reciprocity, appreciation [34, 42, 60, 61, 73], knowledge of the culture [40] and identity, consider time and lived experiences [34, 60], humility, care and generosity [34].	
• **Ethical research** is research that empowers, provides social justice [40], emancipatory, decolonizing, protects [15, 74], gifting, knowing the language or dialect and build capacity [60].	
• **Reciprocity** equitable benefits to indigenous populations, joint ownership [22, 73]	
• **Ontology** of indigenous people is defined as the point of view of spirituality and their interconnectedness to their land and the environment which require a participatory approach to ethical research. **Participatory approach** comprises the ethical principles of reciprocity, respect, equality, responsibility, survival and protection or safety, spirit and integrity, partnership, responsiveness and benefit [15, 22, 23, 40, 42, 60, 73, 94]	
(iii) **Vulnerability and minority status of indigenous populations exploited for research**	
• **Common issues**; marginalization, exploitation and lack of benefits [42, 46, 94].	
• Western paradigms are associated with expert knowledge while indigenous paradigms are associated with “lay knowledge”. **Western ideas adopted in research methodologies cannot** be applied to the understanding of Pacific Island culture [25, 27, 54, 60].	
(iv) **Research Ethics Governance with Indigenous People**	
Principles commonly expressed in national HRE Guidelines are;	
• **Responsibilities and cultural continuity** [28, 64]	
• **Respect for persons** [28, 64, 81]	
• **Spirituality, integrity, equity and justice** [40, 64]	
• **Relationships** [28, 40, 64, 80]	
• **Research designs** are to include the principles of confidentiality, protection of human subjects’ dignity and safety, maximize efficiency, transparency, accountability, fair open and responsible conduct. Improve health and benefits aligned towards national priority areas [40, 80]	
(v) **Research Ethics Committees**	
• **Stewardship roles** within health research systems [36]	
• HREC is **underdeveloped or lacking** [20, 24]..	
• **Capacity building** is needed to develop governance mechanisms. LMICs lack resources, have limited legal systems and little expertise in bioethics. **Capacity building** and institutional support for HRECs is needed yet lacking [20, 24]..	
• HRECs cannot function on **altruistic grounds only** [3].	

### Indigenous and cultural principles of HRE

This section discusses a critical analysis of indigenous principles expressed in literature, including relationship building, and the ontology of indigenous populations in Oceania. Indigenous research principles include respect for relationships, knowledge and reciprocity, and participation among other principles [35, 53, 60, 71, 92]. Indigenous peoples, not unreasonably, expect research to respect indigenous principles at all stages of the research process. Health research institutions have a responsibility to support ethical research involving indigenous persons that will contribute to the elimination of inequity in health [94].

**Relationship building** with community leaders and prospective participants in cross cultural research is very important [57]. Concepts used for relationship building are common among Australian Aboriginal peoples and Torres Strait Islanders [73], Cook Islanders [35], iTaukei, who are the indigenous people of the Republic of the Fiji Islands [60], Maori of New Zealand [61] and Tongans [34]. Relationship building involves the following concepts: respect, empathy [26], collaboration, sharing of resources, reciprocity [42, 60, 61, 73], appreciation [34], knowledge of culture and identity of indigenous people, consideration of time and lived experiences [34, 60].

For a relationship or *Va* in Tongan to begin [57], researchers need to develop respect for indigenous people’s culture, philosophy, environment, spirituality and epistemology [60]. If respect is achieved by the researcher, respect will also be given by the indigenous people because a relationship has been formed. Time and lived experiences are important factors in the relationship building as expressed by [34] as *nofo* in the Tongan context. *Nofo,* “to stay” and “to live”, means that the person has to stay for some time within the indigenous community and *talanoa* (verbal discussion or talk*)* about things or just talk with people at leisure [60, 76, 78, 90, 91]. While *talanoa* takes place, the relationship is being formed. Sharing of resources will also take place and the relationship grows stronger. Farelly and Nabobo-Baba [26] emphasized that *talanoa* as a decolonizing research method ought to reflect the socio-cultural background of the indigenous research setting [26]. The process of t*alanoa* comprises a combination of lived experiences, ecology, imaginations, memory, and body language of persons in the *talanoa* group. Through knowing people in the indigenous community through the relationships established over time, researchers will be assisted by the community groups. Research protocols such as ‘entry’ to indigenous communities will be informed by selected members of the indigenous community. For example, in indigenous Fijian iTaukei, “*Na i curucuru/na i sevusevu*” (entry) is the process performed by the indigenous researcher’s party to ask the community for permission to enter the community in order to conduct a *talanoa* (oral communication) for research purpose or other purposes [60]. Authors recommend that research institutions should aim to align their roles with research approaches appreciated by indigenous communities of Maori and of indigenous origins. Indigenous communities appreciate research that empowers, provide social justice, is emancipatory and supportive of decolonization [15, 76].

Authors expressed two opposing views of indigenous persons and resources in cross-cultural research settings as well as conflicts within indigenous groups. Firstly, the exploitation of indigenous persons and resources in cross-cultural research can happen through the inaccurate reporting of research findings [48]. Secondly, individual members of the community can become greedy and guard access to indigenous resources, including knowledge, gifts or gifting and spiritual blessings [34, 60, 61, 74, 78], in order to commercialize these and make profit for personal gain [33]. These views may be considered as ethical dilemmas formed because of differences in attitudes, habits or dissolutions amongst people concerned [45].

The **ontology of indigenous peoples** including the Aboriginal peoples and Torres Strait Islanders of Australia, iTaukei (indigenous people of Fiji), Maori of New Zealand and the Samoans, is relatively different from the ontology of Western thought. The difference is commonly defined by authors from the point of view of indigenous peoples’ spirituality and their interconnectedness to their land, with a participatory approach to HRE recommended [15, 22, 23, 42, 60, 73, 94]. The participatory approach comprises the ethical principles of reciprocity, respect, equality, responsibility, survival and protection or safety, spirit and integrity, partnership, responsiveness and benefit [15, 22, 23, 42, 60, 73, 94].

Respect in research is ethical research that empowers and protects the indigenous community [22]. In addition, the principle of reciprocity ensures the distribution of equitable benefits to indigenous populations participating in research. An indigenous reference group and joint ownership of research between researchers and indigenous research participants is recommended specifically for Aboriginal and Torres Strait Islanders [22, 73]. Broadly, a respectful research approach for Tongans includes the principles of *feveitokai’aki* (mutual care and generosity) and *loto fakatokilalo* (humility).

Contemporary Samoan experiences and ethical approaches are central within the principles of *tapu* (the sacred) and *tofa sa’ili* (the search for wisdom) [23]. The Vanua Research Framework of Fiji recognizes the importance of gifting and knowing the language or dialect of the research community for accuracy of understanding, critiquing, verifying and documenting indigenous concepts in research. Vanua also recommended that an iTaukei should be the principal researcher for capacity building purposes [60]. Researchers’ accountability means that Vanua chiefs and community must grant permission for all research projects done in the Vanua [42, 60, 61]**.** On the other hand, the operationalization or application methods of the principles of research in an indigenous setting is not well represented in the literature [44].

### Informed consent (IC) in indigenous settings in Oceania

The need for IC is central to ethical research in indigenous settings [6]. The moral values most commonly defined as elements of IC are; (i) a mechanism for respect of dignity and autonomy of persons that should be meaningful, trusting, transparent, un-intrusive, free of coercion, free and informative to protect human rights and bioethics, (ii) collaborative and establishing a trusting relationship [5, 32, 51]. Respect for persons in human research involves the process of voluntary IC, where persons are asked for their permission to participate in a research study [32, 50]. It is a unique and complex process to seek IC among indigenous peoples so it is recommended that a member or members of the indigenous community be involved in the negotiations or consultations about the appropriate method of IC with indigenous communities [32]. Awareness of indigenous culture and language are essential in seeking indigenous persons’ IC [34, 60]. The principle of trust is an essential component of the IC process in any cultural setting. Trust is also significant in research involving indigenous people because trust has the potential to strengthen collaborative relationships in research in any indigenous or cultural setting [50].

An individualistic approach to informed consent involves a competent individual who exercises autonomy in deciding to consent or not. A communitarian approach to informed consent involves the individual and his or her immediate family members, extended family members and the wider community [93]. Leaders of the extended family or communities can anticipate the risks and benefits as persons and as a group in the community before granting informed consent in research [77]. It is recommended that informed consent is to be sought from both individual participants and the local community [32, 54] because the individual is an intrinsic part of the extended family and the community and they are the owners of cultural knowledge that is set within a collective structure [93]. An individual within this community setting does not have the autonomous authority to grant permission through an individual consent process to share and communicate cultural knowledge [93]. The process of IC described as acceptable in indigenous communities includes the use of audio-visual materials and graphics to illustrate concepts and increase understanding. A choice of oral or written IC should be provided for indigenous persons [32, 48, 50]. It is recommended that appropriate processes of IC for research in cross cultural setting include a read aloud session for illiterate people or if participants have diminished autonomy, for example, refugees have diminished autonomy and cannot sign an IC form because identifying them with their names and signatures may pose high risk even death [48].

### Vulnerability and minority status of indigenous populations exploited for research

This section presents incidences of exploitation experienced by indigenous persons or groups in human research. Notions of marginalization, exploitation and lack of benefit for indigenous participants in unethical medical research were commonly expressed [46] [42] [94]. Human research with Aboriginal and Torres Strait Islander population of Australia are described as biased, disempowering and offering inadequate protection [22, 73].

In the Pacific Region, Autogen^2^ received the consent of the Tongan Government, but the Tongans who were the prospective participants in the genetic study did not consent. This is a paternalistic act of the Tongan government. The Autogen proposal was rejected by Tongans because of a lack of public discussion, the wrong approach to informed consent and the religious sanctity associated with the blood of Tongans [9]. The Autogen approach to informed consent in its proposed genetic study in Tonga was individualistic, which was one of the reasons for the rejection of the proposed study by Tongan people [9].

Indigenous epistemologies have been long subjugated, while the application of Western ideologies in indigenous settings seem to have been the norm for many years, for example, the attempt by Autogen to apply an individualistic approach to informed consent in an indigenous communitarian setting like Tonga was a mistake. The outcome of making such a mistake is that the proposed Autogen genetic research in Tonga was cancelled. The Australia Broadcasting Commission (ABC) Pacific Beat reported an American company Phoenix Life Sciences had chosen the Melanesian country of Vanuatu despite its dormant ethics committee, to trial cannabis-derived drugs. ABC reported that Phoenix Life Sciences was forced to move to Vanuatu because of strict drugs legislations in the USA and Vanuatu was attractive because of “the ability to work through the Dangerous Drug Act”. Phoenix Life Sciences move was strongly opposed by the Vanuatu Medical Association [1, 16, 37]. In Guam a 1996 law created the Guam Ethics Commission and was re-established in 2004, but the Guam Ethics Commission has not been active for 23 years and by 2019 it was reported that there were still no appointments to the Ethics commission yet and people of Guam have been waiting for two decades [70].

Western paradigms are associated with expert knowledge while indigenous paradigms are associated with “lay knowledge” [54]. Western ideas adopted in research methodologies cannot be applied to the understanding of Pacific Island culture [25, 27, 54, 60]. A good example is a study proposed by Meo Sewabu in Fiji to involve people with whom she has an existing relationship [54]. This project is considered unethical from the Western point of view because of a presumed lack of objectivity involved in the research methods [54]. An indigenous researcher’s paradigm considers the involvement of indigenous thoughts and methods as fundamental to research and researchers are to comply with indigenous peoples’ expectations [60, 73, 74]. If indigenous methods are excluded, the researcher will face major challenges in the conduct of research [54], like a lack of interest and participation from people in all areas of the research ([34, 60]. Culturally, it is believed that if the researcher is indigenous Fijian, negative cultural impact, like unexplained illness, is believed to befall the person or his or her extended family [54]. The Fijian researcher must be culturally sensitive in conducting research in a Fijian setting [54, 60]. A culturally appropriate protocol to observe is the performance of the ‘*Isevusevu*’, which is a presentation of ‘*yaqona*’ (‘piper methystica’, also known as ‘*kava*’ in some Pacific Island countries) by the research team as a request for entry into a Vanua (home, village or community) in Fijian communities [54, 60]. If the *Isevusevu* is not conducted the people of the Vanua will feel disrespected and will not welcome the research team.

The practical advice offered about the ethical conduct of research in indigenous populations involves the inclusion of internationally accepted ethical principles such as beneficence, non-maleficence, tolerance for ambiguity, patience, adaptiveness, an open mind and courtesy. Risk and benefit analyses and research projects that maximize benefits for indigenous populations should be conducted [45, 48]. Advice to researchers for successful research in indigenous populations is to be willing to learn and have thorough comprehension of the culture [7, 34, 60, 73]. Research participants may be in a position of vulnerability due to a perceived lack of understanding of the purpose of the research and the risks and benefits of participation in the research [6]. It is important to recognize and address power relations and level of knowledge between researchers and research participants in order to reduce their vulnerability due to lack of power relative to the researchers [6].

Researchers are to immerse themselves in the culture of indigenous populations to develop an in-depth and accurate comprehension of the indigenous populations’ culture [5, 34, 42, 73, 94]. Researchers are to also gain extensive historical, socio-cultural and religious background knowledge of the indigenous populations in cross-cultural research [45, 48, 52, 69]. An interesting description of research bioethics training of people from developing countries is “indigenous evangelization” whereby indigenous persons are being taught or “evangelized” by the Western bioethical principles [19] and in the process losing sight of traditional ethical principles.

### Research ethics governance for Oceania indigenous people

Statements for the governance of human research in indigenous populations have been developed in some countries of Oceania region. Some of the statements identified in this scoping literature review were from Australia, Fiji, New Zealand; Pacific statements issued by New Zealand Universities and Tonga. The title and the citation for these statements are presented in Table 4.

**Table 4:** Statements for the governance of human research in Oceania indigenous populations

1. Te Ara Tika: Guidelines for Māori research ethics: A framework for researchers and ethics committee members [40]	
2. Pacific Research protocols from the University of Otago [5]	
3. Pacific Health Research Guidelines [39] 4. Operational Guidelines for the National Health Ethics and Research Committee [81] 5. Fiji National Health Research Guide [28]	
6. *Ethical conduct in research with Aboriginal and Torres Strait Islander Peoples and communities: Guidelines for researchers and stakeholders and Keeping research on track II* (National Health and Medical Research Council [64] 7. *Centre for Samoan Study at National University of Samoa,* University Research and Ethics Committee (UREC), 2020 [12]	

The cases of the Fiji and Tonga HRE governance mechanisms need elaboration. Although there are existing documents about Fijian and Tongan cultural standards and frameworks of human research developed by indigenous Fijians [54, 60] and Tongan scholars [34, 78, 91] respectively, there are no links from these guidelines to the research ethics frameworks to indicate the inclusion of the cultural values of human research in the governance mechanisms of HRE. There may be reasons for the non-inclusion of cultural frameworks in the National Health Research Guidelines in Fiji and Tonga, but those reasons are currently not documented.

An interesting variation was identified in the lists of references of the national guidelines of human research in indigenous populations of high-income countries compared to low and middle income countries. The “*National Statement on Ethical Conduct in Human Research: 2007 (Updated 2018)” in Australia* [65]*, the “Te Ara Tika*: Guidelines for Māori research ethics” [41] and the “Pacific Research protocols” from the University of Otago [5] made reference to both prominent international guidelines such as the Declaration of Helsinki, Belmont Report, Nuremberg Code as well as policy documents that govern indigenous research. These include the National Aboriginal Health Strategy Working Party (1989), and [42]. This is evidence that government policies for governing indigenous people were sourced and included in the processes of developing HRE governance mechanisms in high income countries of Oceania. On the other hand, the national guidelines for low and middle income countries’ such as Fiji and Tonga make reference only to prominent international HRE guidelines. The impact of the variation is reflected in the Guidelines, but there is no mention of cultural or indigenous principles of HRE.

### Research ethics committees in Oceania indigenous populations

Research Ethics Committees (RECs) or Ethics Committee (EC) [36, 41] or Research Ethics Board (REB) [77] or Human Research Ethics Committees (HRECS) [20] are different names of research ethics committees but they all have the same goals which is to govern research in order to protect human participants and to ensure that the research projects benefits, or at least does not harm participants but often benefits of research is not directly for participants [36].

HRECs were established worldwide in the early 1980s with stewardship roles within health research systems. HRECs are well developed and strong in Australia, New Zealand for indigenous people but not so in other countries of Oceania like Fiji. LMICs which includes some countries in Oceania lack resources, have limited legal systems and little expertise in bioethics [24]. Online news media reported that; (i) Vanuatu Ethics Committee has been described as dormant [1] and the Guam Ethics commission has been inactive for 23 years [70].

In a time of increasing health inequity around the world, (HRECs) must provide oversight of human research such as review and approval of human research proposals [18, 36]. A critique of REC relative to indigenous knowledge construction is that the result of ethics review further marginalize indigenous approaches to knowledge construction and dissemination because REBs employ universal and individualized approaches to the review of research involving indigenous populations [77]. REBs are to recognize indigenous communal processes in indigenous research [77].

Capacity building and institutional support for HRECs is needed yet lacking. HRECs cannot function on altruistic grounds only where members’ contributions are for the benefits of others only, but HRECs need funding and support in the form of human resources in order to function effectively [3]. The HRE systems in lower income countries of Oceania could be strengthened by incorporating indigenous principles and practices. Countries in Oceania region that do not have an HRE system need to build capacity in order to develop their human research governance mechanisms. Researchers and ethics committees are to support HRE and maximize the protection of indigenous people in human research [36]. Some Pacific Islands have only informal processes that exist for ethics review and oversight. Cook Islands does not have a HREC and relies on overseas Ethical Review Committee; Tonga has a National Human Research Ethics Committee but do not have regular meetings; Samoa’s HREC was under review and now has a University Research and Ethics Committee in the Centre for Samoan Studies at the National University of Samoa [12]; Vanuatu has some form of ethical review process under the Corporate services of the Ministry of Health [24]. This is problematic for the optimal development of relevant and culturally appropriate research and building up local ethics committees should be part of continued research development in the Pacific [20, 24].

## Discussion

**Fig. 2:**
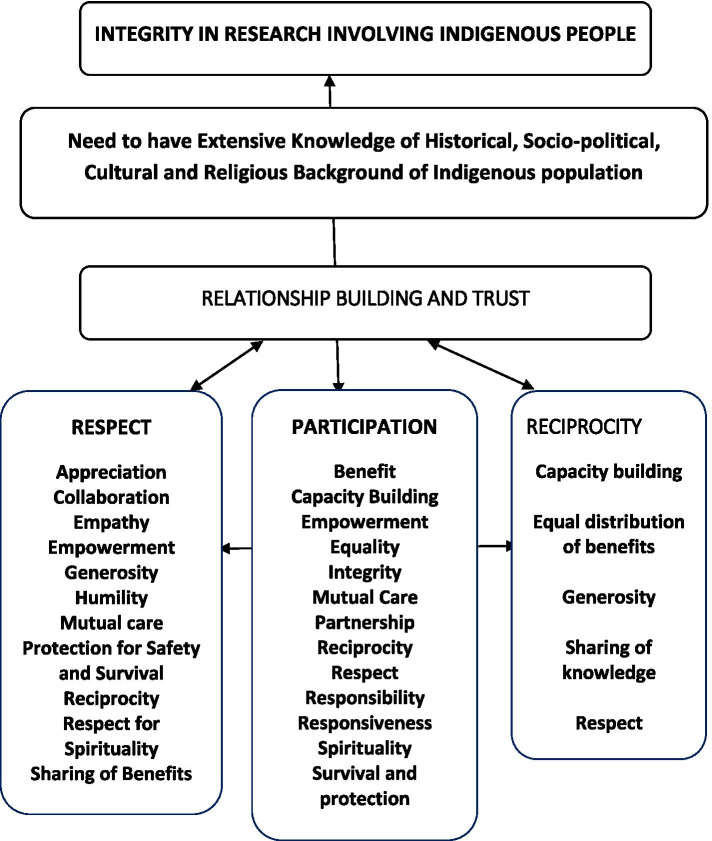
Distinctive HRE Principles common to indigenous populations in Oceania, a mind map

The aim of this scoping literature review is to explore how Oceania regional countries’ indigenous knowledge of HRE is engaged in the governance of research involving human participants. The findings demonstrate significant development in the governance of HRE in developed countries like Australia and New Zealand, for the protection of Australian Aboriginal and Torres Strait Islander indigenous peoples and Maori of Aotearoa (New Zealand). There are existing regulations, policies or guidelines developed solely for the protection of indigenous people in human research in Australia and New Zealand. The guidelines inform researchers of indigenous principles and methods appropriate for the conduct of research with indigenous people. By contrast, this is not the case in countries like Fiji, Tonga, Vanuatu and other PICs. For researchers to achieve integrity in research that involves indigenous people, they need to gain extensive knowledge of indigenous history, socio-cultural and religious backgrounds before starting a research project. Distinctive HRE principles and values identified in this review that are common threads in research settings with Oceania indigenous populations are mapped in the flow diagram (Fig. 2).

The engagement of indigenous principles in HRE regulations and guidelines ensures the achievement of research integrity. Two HRE guidelines, (i) Values and ethics: Guidelines for ethical conduct in Aboriginal and Torres Strait Islander health research [66] and (ii) Te Ara Tika: Guidelines for Māori research ethics: A framework for researchers and ethics committee members have set the precedent for inclusion of indigenous peoples’ principles in HRE guidelines for countries of Oceania. Various other guidelines from New Zealand Universities identified as “Pacific” were also identified to include cultural principles of human research [5, 55]. It is, however, different for small nations where the majority of people are indigenous, for example, Fiji and Tonga. Fiji’s iTaukei population and the Tongans in Tonga hold are majority population, but have not developed national HRE guidelines to include indigenous and cultural values and principles. The indigenous principles and values and their core elements will foster development of a mutual relationship between researchers and indigenous participants in research.

It is interesting to note the portrayal of the indigenous principles of ‘respect’ in literature reviewed. Respect described by authors as encompassing the values of mutual care, humility, empathy, collaboration, generosity and sharing of benefits, reciprocity, appreciation, awareness of culture and languages, protection, safety, survival and spirituality. Respect and its core values are highlighted in the literature thus respect is established as one of the foundational pillars of relationship building and trust in research with integrity involving indigenous populations. Indigenous peoples demand the application of respect through various methods in research to uphold their dignity and right.

The notion of ‘participation’ encompasses partnership, benefits, capacity building, empowerment, equality, integrity, mutual care, partnership, reciprocity, respect, responsibility, responsiveness, spirituality, survival and protection. Empowerment of all stakeholders in research emerges via ‘participation’. Capacity building involves indigenous people’s involvement in the conduct of research projects with a goal of increased knowledge of conducting research. Capacity building can also be a two-way learning experience where everyone learns and teaches. Reciprocity in research involving indigenous people applies to respect, sharing of knowledge among all stakeholders, being generous and equal distribution of benefits among all stakeholders and capacity building.

An interesting reciprocal angle of empowerment via ‘participation’ emerges. Where there is participation of indigenous people in research, ‘doors’ into indigenous knowledge and societal etiquettes will open to research stakeholders through indigenous participation. This reciprocity in participation enables a successful research endeavour, which will bring benefits to all parties involved. The notion of “reciprocity” further emphasizes equality in the distribution of benefits of research. Benefits that are both tangible and intangible should be shared equally among research stakeholders and be enforced via HRE regulations.

## Conclusion

This scoping literature review has significant findings. Relationship building between researchers and indigenous populations is key to a successful research in indigenous populations. HRE principles and their elements important for relationship building include respect that is reciprocal between researchers and indigenous people. Elements of the principle of respect are highlighted, including empathy, collaboration, sharing of benefits, reciprocity, appreciation, empowerment, protection for safety and survival, respect for spirituality, and awareness of culture and languages. The ontology of indigenous people is different from the ontology of Western thoughts. Indigenous ontology as understood for research includes spirituality, connectedness to land, religious beliefs and a participatory approach to HRE. The participatory approach comprises the ethical principles of reciprocity, respect, equality, responsibility, survival and protection or safety, spirit and integrity, partnership, responsiveness and benefit. Western paradigms adopted in research methodologies can create challenges in indigenous settings because of the differences in paradigms. Indigenous paradigms and applications methods are fundamental to research involving indigenous populations. A participatory approach in an informed consent process would be first step to build a trusting relationship between researcher and research participants.

The findings indicated the need to make recommendations for successful research involving indigenous populations in Oceania. Indigenous HRE principles and applications methods should be part of HRE governance mechanisms. Informed consent processes in indigenous settings should be informed by both individualistic approach and a communitarian approach. The existing research frameworks in countries of Oceania could be linked and highlighted in HRE governance mechanisms. Capacity building and institutional support for the establishment of Research Ethics Committees is needed. Governance mechanisms of RECs, once established, must incorporate indigenous principles and applications in order to maximize the protection of the dignity and rights of indigenous peoples of Oceania.

## Data Availability

Not applicable.

## Abbreviations

HIC: High Income Country
HRE: Human Research Ethics
HREC: Human Research Ethics Committee
IC: Informed Consent
LMIC: Low and middle income countries
PIC: Pacific Island Country
PRISMA: Preferred Reporting Items for Systematic Reviews and Meta-Analysis
REB: Research Ethics Board

## Footnotes

1 Tonga does not fit the definition of indigenous as per [58], because Tonga is a Polynesian Kingdom that was never colonized and do not recognize themselves as indigenous.

2 A proposal by a Melbourne based biotechnology company, Autogen, to establish a database of genetic information on the population of Tonga. The project proposed by Autogen in Tonga in the South Pacific was opposed by Tongan church groups and pro-democracy groups. Autogen emphasized individual informed consent but the Tongan people’s representatives said that informed consent from the extended family should also be considered because of the common genetic material from extended family BURTON, B. 2002. Proposed genetic database on Tongans opposed. *BMJ: British Medical Journal,* 324**,** 443–443.
